# Two-step crystallisation in a 2D active magnetic granular system confined by a parabolic potential

**DOI:** 10.1038/s41598-023-35135-2

**Published:** 2023-05-26

**Authors:** A. Escobar, M. Ledesma-Motolinía, J. L. Carrillo-Estrada, F. Donado

**Affiliations:** 1 Instituto de Ciencias Básicas e Ingeniería, Universidad Autónoma del Estado de Hidalgo-AAMF, Pachuca, 42184 Mexico; 2 Instituto de Física “Luis Rivera Terrazas”, Benemérita Universidad Autónoma de Puebla, Puebla, 72570 Mexico

**Keywords:** Physics, Statistical physics, thermodynamics and nonlinear dynamics, Phase transitions and critical phenomena

## Abstract

We studied the two-step crystallisation process of a magnetic active 2D-granular system placed on different lens concaveness and under the action of an alternating magnetic field which controls its effective temperature. We have observed that the two-step features of the crystallisation process are more evident as the depth of the parabolic potential increases. At the initial formation of the nucleus, as a first step, in the central region of the lens an amorphous aggregate is formed. In an ulterior second step, this disordered aggregate, due to the effective temperature and the perturbations caused by the impacts of free particles moving in the surrounding region, evolves to an ordered crystalline structure. The nucleus size is larger for deeper concaveness of the parabolic potential. However, if the depth of the parabolic potential exceeds a certain value, the reordering process of the second step does not occur. The crystal growth occurs similarly; small disordered groups of particles join the nucleus, forming an amorphous shell of particles which experiments a rearranging while the aggregate grows. In the explored range of the depths of the parabolic potential, crystallisation generally occurs quicker as the deeper parabolic potential is. Also, aggregates are more clearly round-shaped as parabolic potential depth increases. On the contrary, the structures are more branched for a smaller depth of the parabolic potential. We studied the structural changes and features in the system by using the sixth orientational order parameter and the packing fraction.

## Introduction

Non-classical nucleation theories arise as alternatives to describe crystallisation in various systems for which classical nucleation theory (CNT) fails^1–4^. According to CNT, particle concentration inhomogeneities lead to the formation of the nucleus in just one step, from which the crystal grows. Starting from a fluid-like state, as the effective temperature diminishes, unstable aggregates form and become larger as the effective temperature decreases^5–7^. For specific conditions, aggregates reach a critical size when free bulk energy surpasses the free surface energy. At this point, the aggregate becomes a stable nucleus and grows to form the crystal. Then, to form a nucleus, it is necessary to overpass an energy barrier. CNT assumes that the nucleus has been an ordered structure since its generation. This theory has successfully described nucleation and crystal growth in some systems but fails in others. For instance, it fails to predict the critical size of the nucleus and the height of the energy barrier, among other quantities that do not correspond to what is observed in some systems^8–10^. Therefore, there is a search for a theory capable of describing nucleation in those systems for which CNT fails.

In one of the non-classical nucleation theories^11,12^, specifically the two-step nucleation theory, it is predicted that an amorphous aggregate is initially formed in the first step. Then it is ordered in the second step^13^. Experimentally, it has been found that in order to occur the first step, the barrier energy that needs to be over-passed is lower than that predicted by the CNT. After that, the system must overpass a second energy barrier to reorder the aggregate to become the nucleus. In this second-step process, thermal perturbations and the interaction of the aggregate with the free particles are crucial in the reordering process that should be detailedly understood^14^.

Some experimental results^15,16^ and numerical simulations^17,18^ support this two-step non-classical nucleation theory. However, due to the microscopic nature of the crystallisation processes, direct experimental evidence describing the phenomena at the particle level is hard to find in the literature. Therefore, it is desirable to contribute to understanding these phenomena with experimental evidence at the particle level, where each particle is followed as it slows down its dynamics while the system diminishes its effective temperature. In particular, it is interesting to determine how the nucleus is formed and how the crystal grows. Information at the particle level would allow us to describe a system’s local dynamics and structural characteristics.

Determining the local dynamics is essential because obtaining averaged quantities, such as those obtained from standard material characterisation techniques, hides these inhomogeneities in systems with inhomogeneities, and we could misdescribe the system. In this context, macroscopic models based on colloidal and granular systems could shed light on some aspects of the crystallisation process because their dynamics are slow, and the sizes of particles are macroscopic. Of course, there are many limitations in this scaling. However, the great advantage of this approach is that we can track the particle behaviour and, consequently, the local structural evolution^19–21^.

Previously, we have reported studies on glass transition, crystallisation process, aggregation, and active matter using a nonvibrating granular system as a macroscopic model for processes observed in fluids^22–28^. In this system, each particle takes energy from the alternating magnetic field and transforms it into kinetic energy, becoming a stochastic self-propelled particle. Each particle in the system can be tracked, letting us monitor the dynamic and structural properties in real time. Each particle has its own stochastic motion independent of the other particles in the low particle concentration regime, provided they are far enough to neglect the dipolar interactions. If particles become closer than in low particle concentration cases, they are complexly coupled, and different phenomena merge as a natural consequence.

In the present crystallisation study, we will show, through a series of experiments, that in systems confined by a parabolic potential, crystallisation phenomena occur in two-step processes. For our experimental setup, we use an observation cell with particles settled in lenses of different focal lengths, i.e. different depths of the parabolic potential. We have observed that parabolic potential makes particles aggregate in the centre of the lens; as the depth is more prominent in a lens with higher curvature, the tendency of the particles to aggregate is more significant. Thus, it is expected that the depth of the potential parabolic to have a noticeable effect on the crystallisation process. We characterised the system employing the sixth orientational order parameter and packing fraction.

## Results

**Figure 1:**
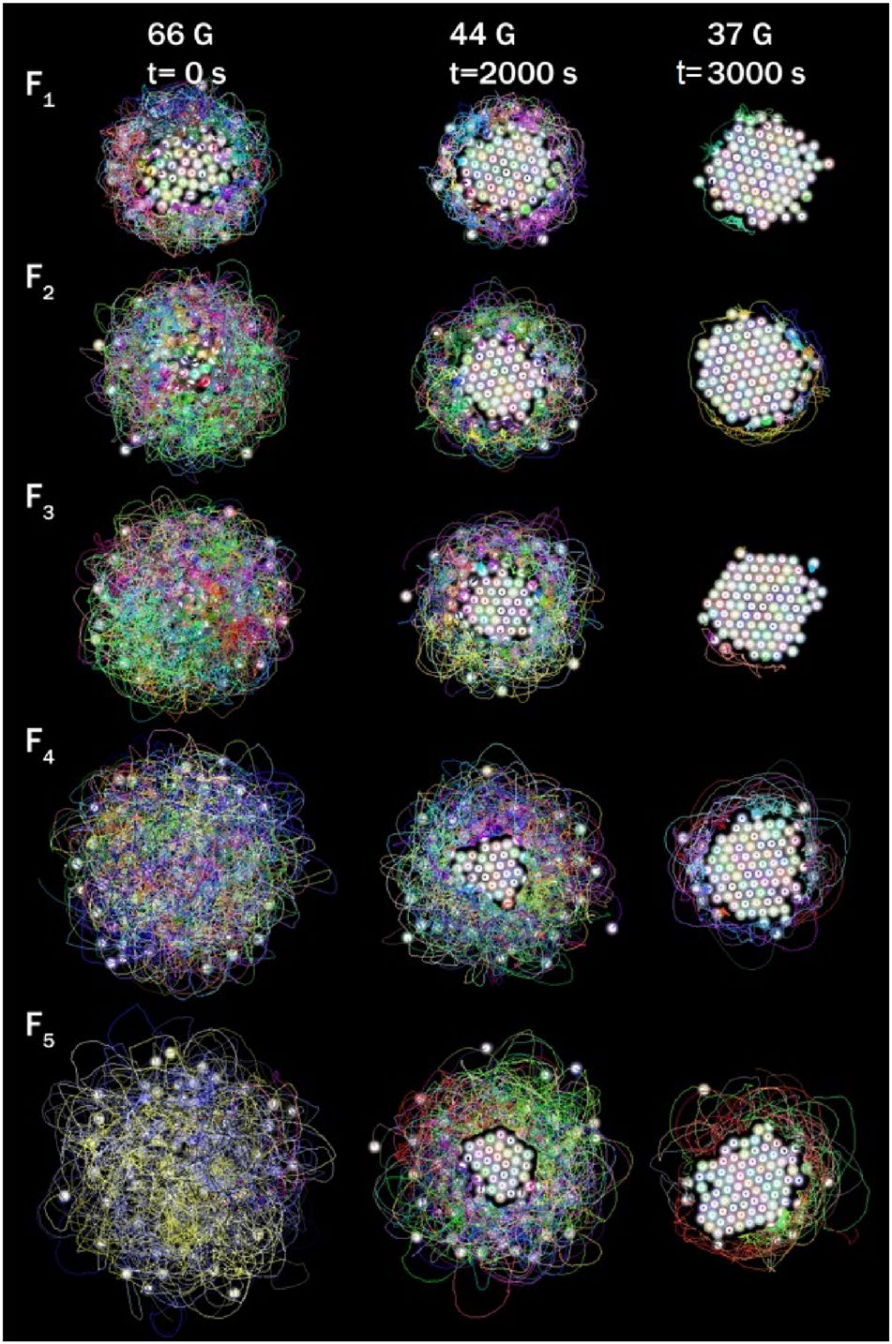
Particle trajectories are observed for the different focal length lenses at three stages, or effective temperatures of the cooling process, high 66 G, medium 44 G, and low 33 G.

The system comprises 90 magnetised spherical steel particles of 1 mm in diameter, $\sigma$, (ANSI 420 grade 1000 by Gimex S.A) settled on a plano-concave lens. The system is in the middle of two coils in a Helmholtz configuration which produce a vertical oscillatory magnetic field. The coils are fed by a Kepco BOP 36-6 M power amplifier controlled by a sinusoidal signal, $B_{0}=A(t)\sin {(2\pi ft)}$, whose amplitude begins at $A(t=0)$= 66.24 G and decreases linearly at a rate of 0.01 G/s, keeping fixed the frequency at *f*=9.24 Hz. By decreasing the magnetic field amplitude, the effective temperature also decreases. Previously, we have demonstrated that the amplitude of the magnetic field is proportional to an effective temperature^22,23^. Thus, here we used *B* as the effective temperature. A detailed description of a similar experimental setup can be seen in Ref.^27^.

Applying the alternating magnetic field produces a random motion of the steel particles according to the following mechanism. When the magnetic field changes direction due to the sinusoidal signal, the magnetic moment of each particle tries to align with the magnetic field. Because of the particles’ spherical shape and their neutral equilibrium, they rotate independently in a random vertical plane to try to reach aligning the magnetic field. Due to the particles being in contact with a surface and not slipping, the particles roll, acquiring kinetic energy. Depending in a complex way on the magnetic field amplitude, the frequency, the particle magnetic moment, and the friction, when the magnetic field changes direction again, a particle continues rolling in the same direction or changes direction. As time passes, the motion becomes quickly random^22,27^. A CCD video camera records the motion of the particles at 30 fps in AVI interlaced format, and a deinterlace filter is applied to increase the time resolution to 60 fps. ImageJ and its Mosaic plugin^29,30^ were used to track and obtain particle trajectories.

We carried out a series of experiments using the different concave lenses of 50.8 mm in diameter and − 100 mm, − 125 mm, − 150 mm, − 200 mm, and − 250 mm focus length *f*; these lenses and their associate experiment are labelled as $F_1$, $F_2$, $F_3$, $F_4$, and $F_5$, respectively. The curvature of the lens, $\kappa$, increases as the focal length decreases, according to the relation $\kappa =\frac{1}{2F}$. Figure 1 shows the particle trajectories for the different experiments at three effective temperatures, high 66 G, medium 44 G, and low 37 G. In the lens, with the higher $\kappa$ value, $F_1$, it is observed that even at this initial high effective temperature, there is already an initial aggregate so that the free particles move only in the periphery. In contrast, the particles move over the entire surface in the lens with the lowest $\kappa$ value, $F_5$. As the effective temperature decreases, the central aggregate grows, covering a larger area. Finally, one observes in each case that the centrally ordered aggregate reaches a compact hexagonal arrangement at low effective temperatures, with only a few particles that could be in disordered positions.

**Figure 2:**
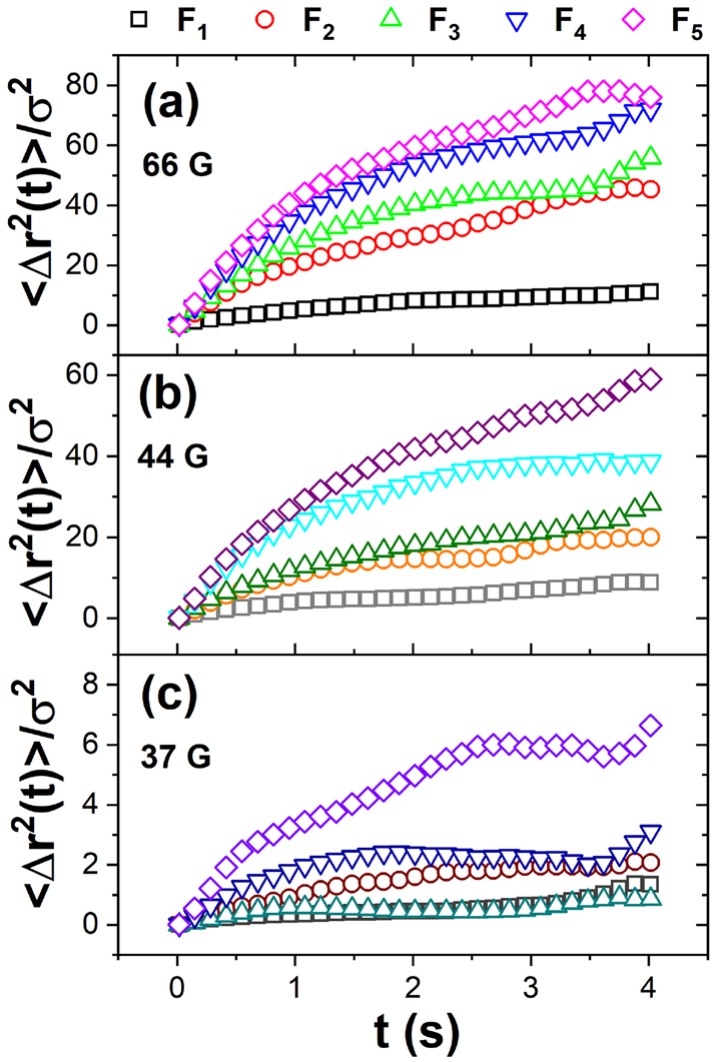
The mean square displacement as a function of time at the initial time (**a**), intermediate (**b**), and immediately prior to the end of the experiment (**c**).

The mean square displacement, $<\Delta {r^{2}}(t)>=<|r(t)-r(0)|^2>$ was calculated from the experimentally determined particle’s trajectories; $\Delta r$ is quantified in particle diameter units $\sigma$. $<\Delta r^{2}(t)/\sigma>$ was obtained for various amplitudes of the applied magnetic field, i.e. effective temperatures. Since the experiments were performed with a linear cooling ramp, the initial, intermediate, and final times correspond to high, medium, and low effective temperatures. Figure 2 shows the time evolution of $<\Delta r^{2}(t)/ \sigma>$ for three temporal stages of the crystallisation process, initial (a), intermediate (b), and long time (c) for all lenses.

**Figure 3:**
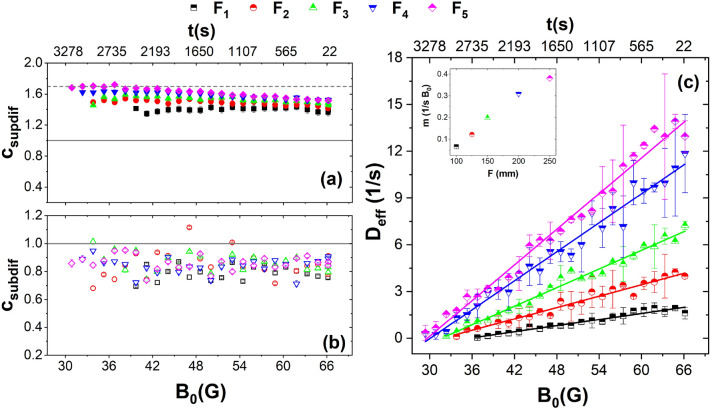
(**a**) Exponent *c* of the power law at the short-time regime or superdiffusive regime, (**b**) subdiffusive regime of the mean square displacement and (**c**) the diffusion coefficient, $D_{eff}$ as a function of the effective temperature. Inset: slope of linear adjustment of the diffuse coefficient as a function of lens curvature.

The range from 0 to 0.1 s of $<\Delta r^{2}(t)/\sigma>$ correspond to the superdiffusive motion, as it is revealed by a power law adjustment $\Delta r^{2}(t)/\sigma>$ $\propto t^c$, where it is observed the exponent *c* shows values around 1.6^31,32^. Whereas the value of the exponent *c* in the range from 0.11 to 0.83 s is very close to the diffusive regime, the exponent *c* shows values around 0.8. Figure 3a,b show the exponent *c* as a function of the effective temperature for the superdiffusive and subdiffusive regimen, respectively. The dashed line corresponds to $c \sim 1.7$, characteristic of active matter agents, and the line corresponds to $c=1$, characteristic of the diffusive regime. The effective diffusion coefficient, $D_{eff}$, was calculated by means of a linear fit of the $<\Delta r^{2}(t)>$ at the same points of Fig. 3b, making use of the relation $<\Delta r^{2}(t)>\sim 4D t$. Figure 3c shows $D_{eff}$ as a function of the effective temperature for the lenses. At high temperatures, the particles move quickly; however, when the effective temperature decreases, the number of free particles decreases, causing a decrease in the effective diffusion coefficient in each of the five lenses we used. This difference in $D_{eff}$ between the initial state (high effective temperatures) and the final state (low effective temperatures) is more evident as $\kappa$ decreases. The rate at which $D_{eff}$ decreases was obtained through a linear fit, represented in the inset as a function of the focal length of the lenses.

**Figure 4:**
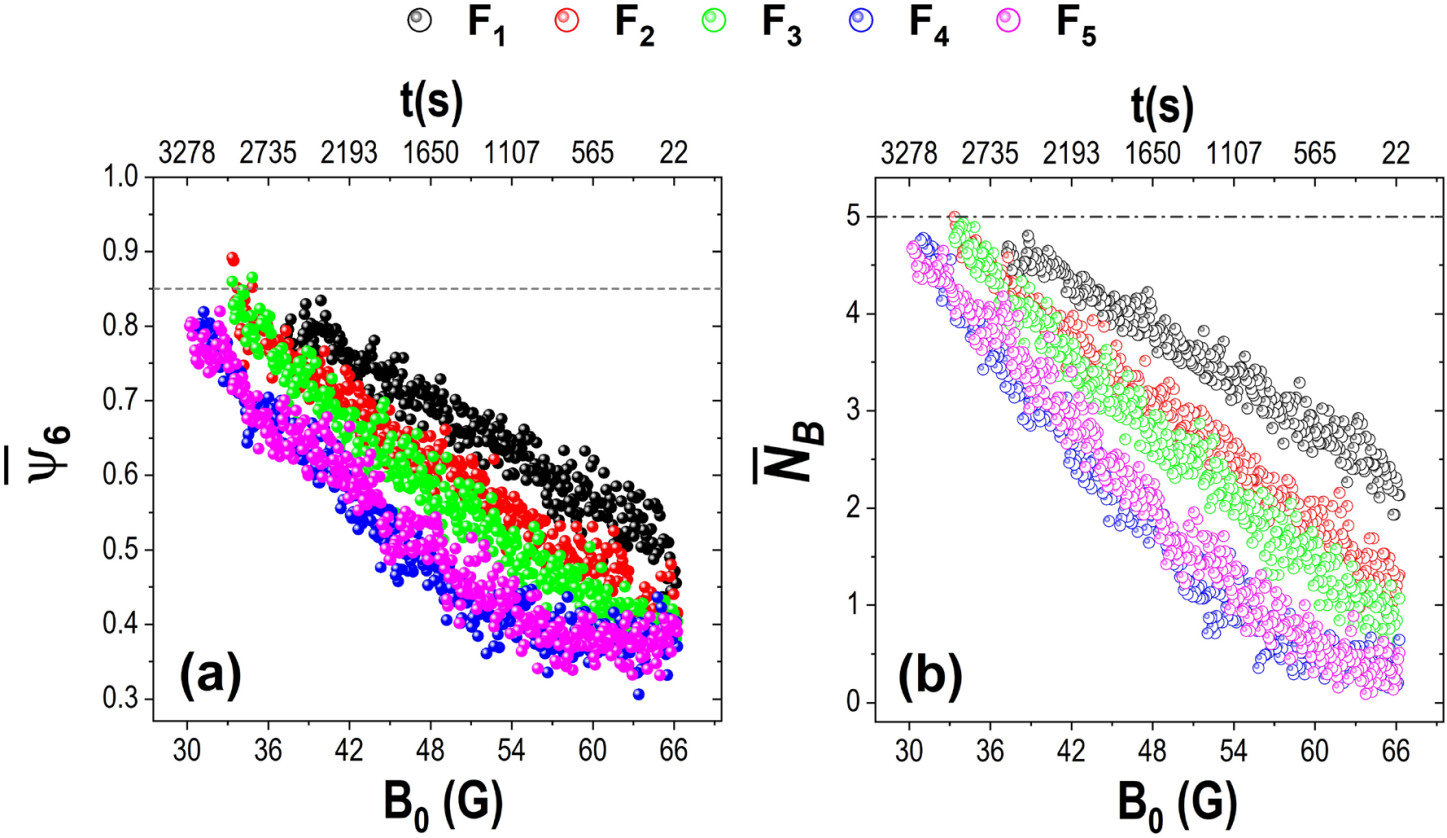
(**a**) Average sixth order orientational parameter, $\overline{\Psi _{6}}$, as a function of the effective temperature, (**b**) the average number of linked neighbours, $\overline{N_B}$, as a function of the effective temperature.

We have found that for a given particle *i*, the sixth order orientational parameter ${\Psi _{6}}$ is a suitable quantity to evaluate the degree of the local order of its closest neighbours, $N_i$^26,33^. Details on how to determine this parameter are discussed in “Methods” section. Figure 4 shows the average sixth order orientational parameter $\overline{\Psi _{6}}$; it allows us to determine, globally, to what extent a particle configuration approaches the hexagonal symmetry. Suppose the value of $\overline{\Psi _{6}}$ is close to unity; in that case, the particle configuration has formed a hexagonal arrangement. In contrast, if the average is far from the unity, the configuration is amorphous or not close to a hexagonal arrangement.

When plotting $\overline{\Psi _{6}}$ for each of the cases we analysed, it is observed that at the beginning of the cooling (66 G), the values are more dispersed and oscillated between 0.35 and 0.6. As the amplitude of the magnetic field decreases, the average of the orientational order parameter approaches the unity. The highest value reached by $\overline{\Psi _{6}}$ is approximately 0.9 since the peripheral particles were not fully aggregated. For the lens with the highest curvature, $F_1$, at the beginning of the cooling, the particles become aggregated disorderly in the lens centre and then progressively become ordered so that the growth of the aggregate occurs in a linear trend. The curves’ behaviour is similar for the lenses, $F_2$ and $F_3$. Most particles move over the lens’s surface, so the $\overline{\Psi _{6}}$ values are below 0.5. Around 60 G, a linear increase of $\overline{\Psi _{6}}$ is observed until it reaches a 0.85 value, where most particles are allocated in minimum energy positions. At the beginning of cooling particles, kinetic energy is high in the cases of the lenses with smaller curvature ($F_4$ and $F_5$). Therefore, their diffusivity is bigger; thus, the orientational order parameter is below 0.4. The aggregation process started below 50 G, and once the central agglomerate becomes stable, the $\Psi _{6}$ values start increasing. Particles achieve a crystalline arrangement of around 33 G.

**Figure 5:**
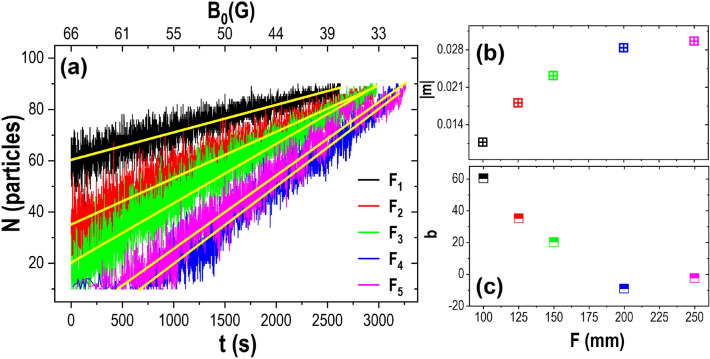
(**a**) Number of particles forming part of the aggregate, a linear fit was made to each of the growth curves, (**b**) the slope of the linear fit, and (**c**) the intersection of the fit with the y-axis as a function of the focal distance of lenses.

Additionally, we evaluate the average number of bonded neighbours $\overline{N_B}$, $N_B$ is the number of neighbours in contact with a given particle, i.e. the coordination number (Fig. 4b). For this, from the nearest neighbours given by the Delaunay triangulation (see “Methods” section), we only count those within a radius 1.1$\sigma$. The behaviour of the average coordination number has a trend very similar to that of $\overline{\Psi _{6}}$. As expected, at the beginning of the experiment, the number of near neighbours was almost zero for the lenses with lower curvature ($F_4$ and $F_5$). The number of bonds increases when $\kappa$ increases; therefore, the interaction between the particles is also higher. For the $F_2$ and $F_3$ lens cases, there is more than one bond for each particle from the cooling start. $\overline{N_B}$ is even larger for the $F_1$ case, where the average $\overline{N_B}$ exceeds the two neighbours. As the effective temperature of the system decreases, particles increase their tendency to aggregate, and the number of bonds increases with time. In a compact hexagonal array, the coordination number of each particle is six. However, when calculating the average number of bonds, the particles dwelling at the perimeter are included; these have two to three neighbours. Consequently, on average, the highest number of bonds is around five.

The number of particles *N* forming part of the aggregate was plotted against time, and free particles were excluded (Fig. 5a). It is observed that once the initial aggregate is formed, the particles continue adhering, and the aggregate grows linearly. However, there are essential differences among the growing processes of the aggregates in each of the lenses. It can be seen that the slope obtained from the linear fits increases as the curvature $\kappa$ decreases (Fig. 5b). The lower the absolute value of the slope, the quicker the crystallisation process occurs. The stronger gravitational potential promotes the accumulation of particles in the centre of the lenses, which promotes nucleation at higher effective temperatures and subsequent crystalline ordering. As the lens curvature decreases, the central aggregate comprises fewer particles, and lower effective temperatures are needed to form a nucleus. For the $F_4$ and $F_5$ lens cases, the effective interaction among the particles was minimal at the beginning of the experiment, and the particles moved randomly over the whole observation area. The aggregation occurs when the temperature has dropped sufficiently; the last two curves start later. Figure 5c shows the interceptions of the linear fittings of the growth curves with *Y*-*axis*. It is observed that as focal distance increases, the initial aggregate decreases. Negative values mean no aggregate is formed at the beginning, and the system must reach a lower effective temperature before nucleation and crystal growth occur.

**Figure 6:**
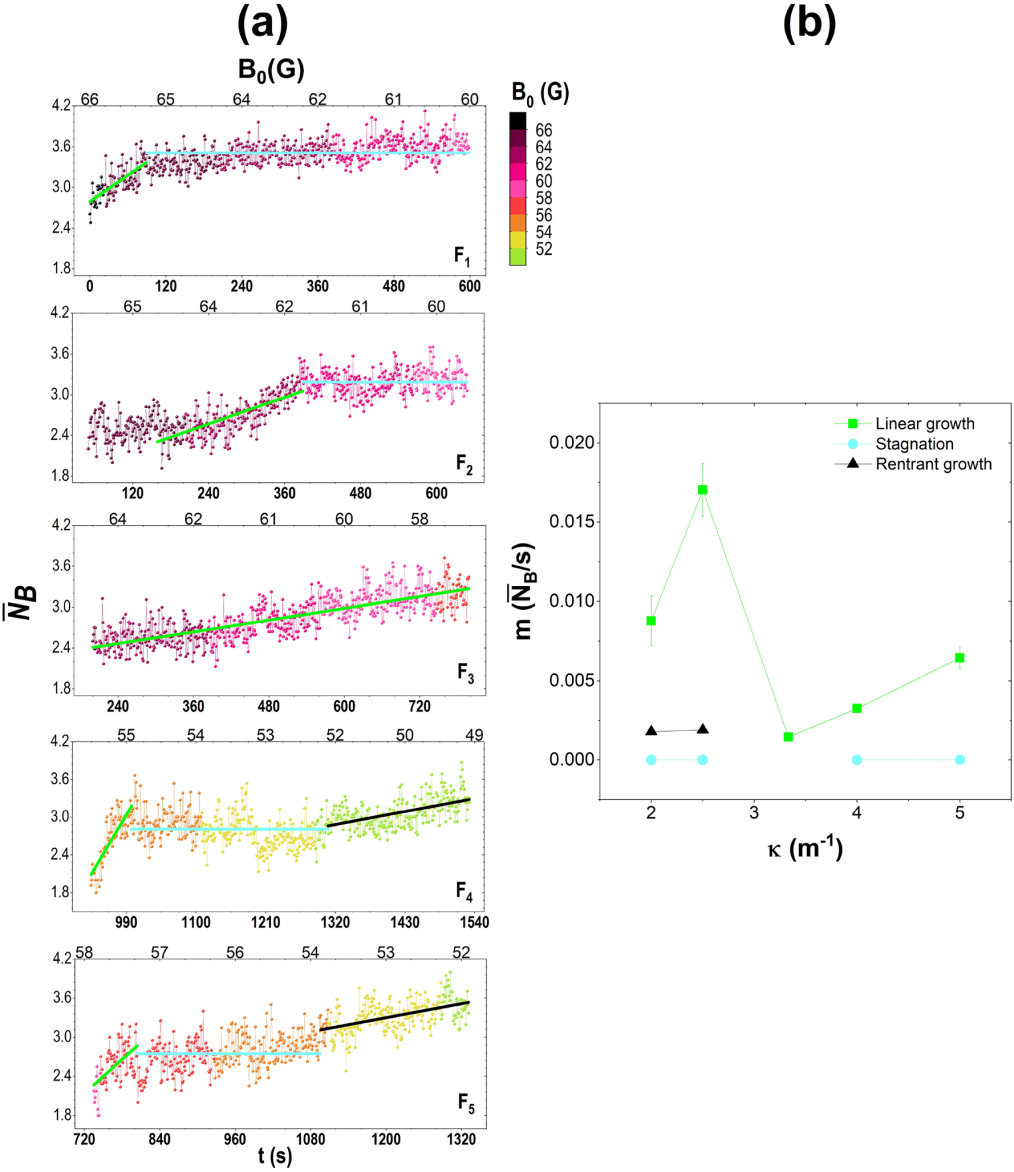
(**a**) The average number of bonds, $\overline{N_B}$ for all lens cases. For this calculation, only the particles that are part of the initial aggregate were taken into account, and $\overline{N_B}$ is calculated from the moment the nucleus is formed until 600 s have elapsed. The green and black lines correspond to linear fits in regions with an increment in the average number of bonds among the particles. The blue line represents a slow growth in the number of bonds, described as stagnation. (**b**) Slopes of the fits as a function of $\kappa$, for the stages of the average number of bonds: linear growth (red), stagnation (blue), and reentrant growth (black).

Now, we will describe the structural characteristics of the aggregates along with the first 10 min from the aggregation beginning for each case. Figure 6a shows the average number of bonds $\overline{N_B}$ of the particles forming the initial aggregate. In each case, our analysis started when aggregation began, and it followed, taking a frame every second. First, one can observe that the aggregation of the particles occurs at different times in each lens. In these curves, one observes all o or some of the following stages: linear and quick growing, indicating the lapse of forming a stable aggregate; stagnation stage, in this, the average number of contacts between the particles remains practically constant; and a linear reentrant growth, that drives the aggregate to a more ordered configuration. In the $F_1$ case, the particles are agglomerated from the beginning of the cooling with $\overline{N_B}$ greater than 2.5. Then it is observed that the linear, quick growing during the next 70 s, the average binding increases to 3.5. Then it is observed the stagnation stage during the remaining seconds. In the $F_2$ case, similar behaviour is observed in the case *F*1. In the $F_3$ case, only linear growth is observed. The three stages can be observed for the $F_4$ and $F_5$ cases. The average number of contacts between the particles was less than 1.8, and after 60 s, the average $\overline{N_B}$ reached the value of 3. From here, there is an interval where the number of interactions fluctuates until 400 s; after that, $\overline{N_B}$ exceeds 3.5 bonds. A coloured bar on the right side of the graph indicates the effective temperature and is indicated on the upper axis of each graph.

Each stage is shown in green, blue and black fitting curves for linear growth, stagnation and reentrant growth. The slopes of these fittings as a function of $\kappa$ are shown in Fig. 6b, where it is observed that the initial growth stages are higher for low values of $\kappa$.

**Figure 7:**
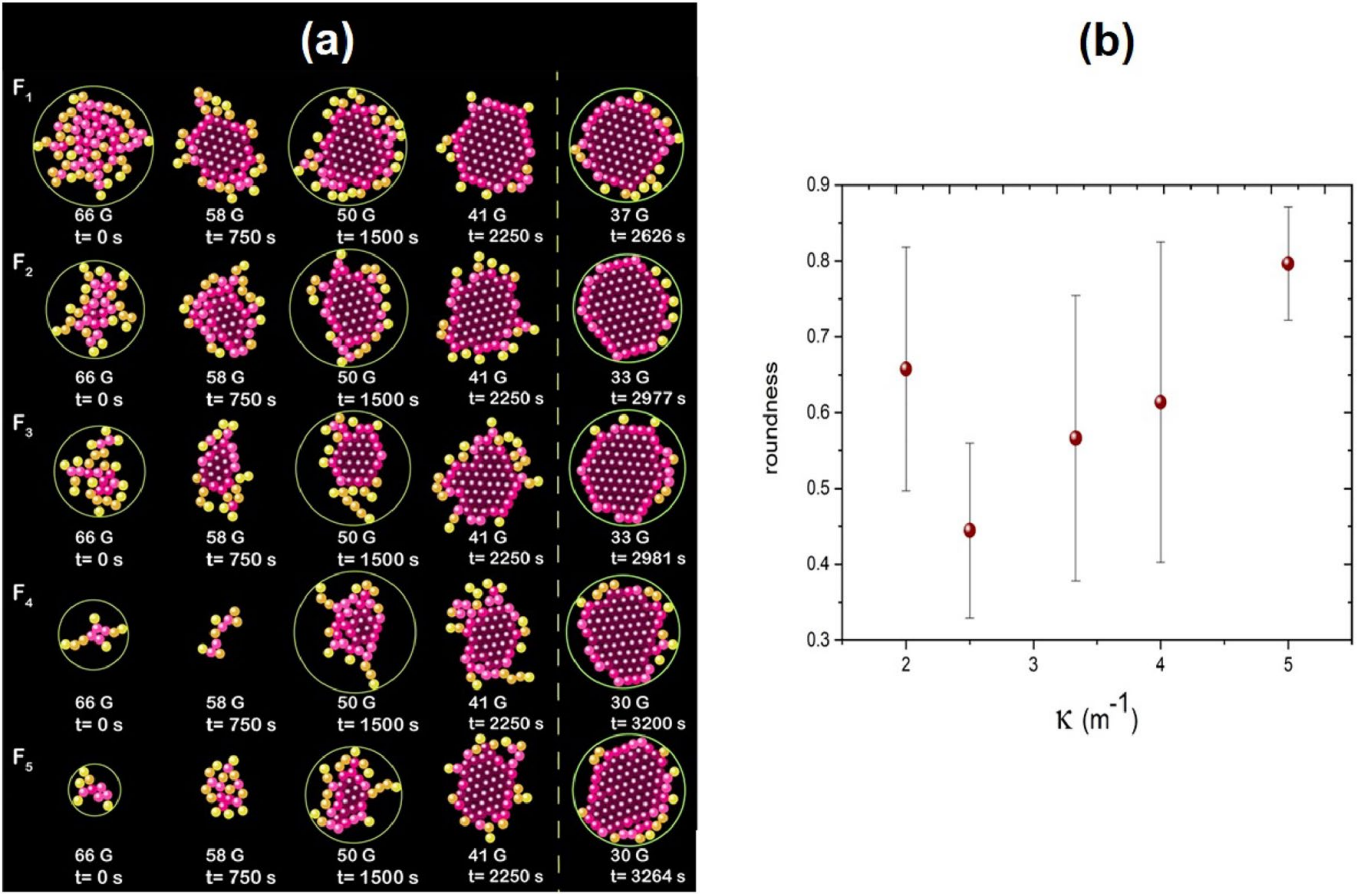
(**a**) Aggregate growth at different effective temperatures for each lens. The comparison is made at the same temperature except for the last arrays that correspond to the final structures since the time of the crystallisation process depends on the curvature of the lenses. (**b**) Roundness as a function of the curvature of the lens, $\kappa$.

The effect of the curvature is evident in the initial configurations. For the highest values of $$\kappa $$ the particles are more in contact and form agglomerates earlier than for the lowest $$\kappa $$ cases. For these latter cases, the initial aggregation is branched due to the dipole–dipole interactions that propitiate the formation of chains. However, once the aggregates grow, the branching decreases. Figure 7a shows particle configurations at different effective temperatures. The first column shows configurations at high temperatures (66 G–50 G). Notice that depending on the strength of the parabolic potential, different cohesive interactions among the particles are produced, causing the cell’s centre to form structures from more to less rounded. As the effective temperature decreases, the particles form a compact hexagonal arrangement in the centre, while particles are disorderly in the periphery. However, the structures are in hexagonal close-packed arrangements at low effective temperatures, 37 G for $$F_1$$ or 30 G for $$F_5$$. This fact is reflected in the average sixth orientational order parameter, $$\overline{\Psi _{6}}$$ presented in Fig. 4 since it reaches the same value for all lenses. To quantify and compare the differences of the configurations in terms of an asphericity parameter as a function of $$\kappa $$, we determined the average roundness of the first ten stable configurations formed during the nucleus’s birth. The roundness is defined as 4*Area/$$\pi $$(mayor axis$$)^2$$. Figure 7b shows the roundness as a function of $$\kappa $$. As the $$\kappa $$ increases, the roundness also increases. Thus, the higher the effective pressure, the shapes are more rounded. In contrast, more branched forms are formed for lower effective pressure.

## Two steps crystallisation

Two-step processes of nucleation and crystal growing are observed in the cases we studied. A stable and amorphous aggregate formation in the lens’s centre is first observed in the nucleation process. Its size and the time formation depend on the $\kappa$ value. In the second step, resulting from the effective temperature and the perturbations caused by the impacts of free particles moving in the surrounding region, it evolves to an ordered crystalline structure. We have also observed that the crystal growing occurs similarly; small disordered groups of particles join the nucleus, forming an amorphous shell of particles which experiments a rearranging while the aggregate grows. Now we will discuss these processes in detail in terms of the populations of particles with five and six neighbours for the formation of the nucleus and the packing fraction in different concentric regions for crystal growth.

**Figure 8:**
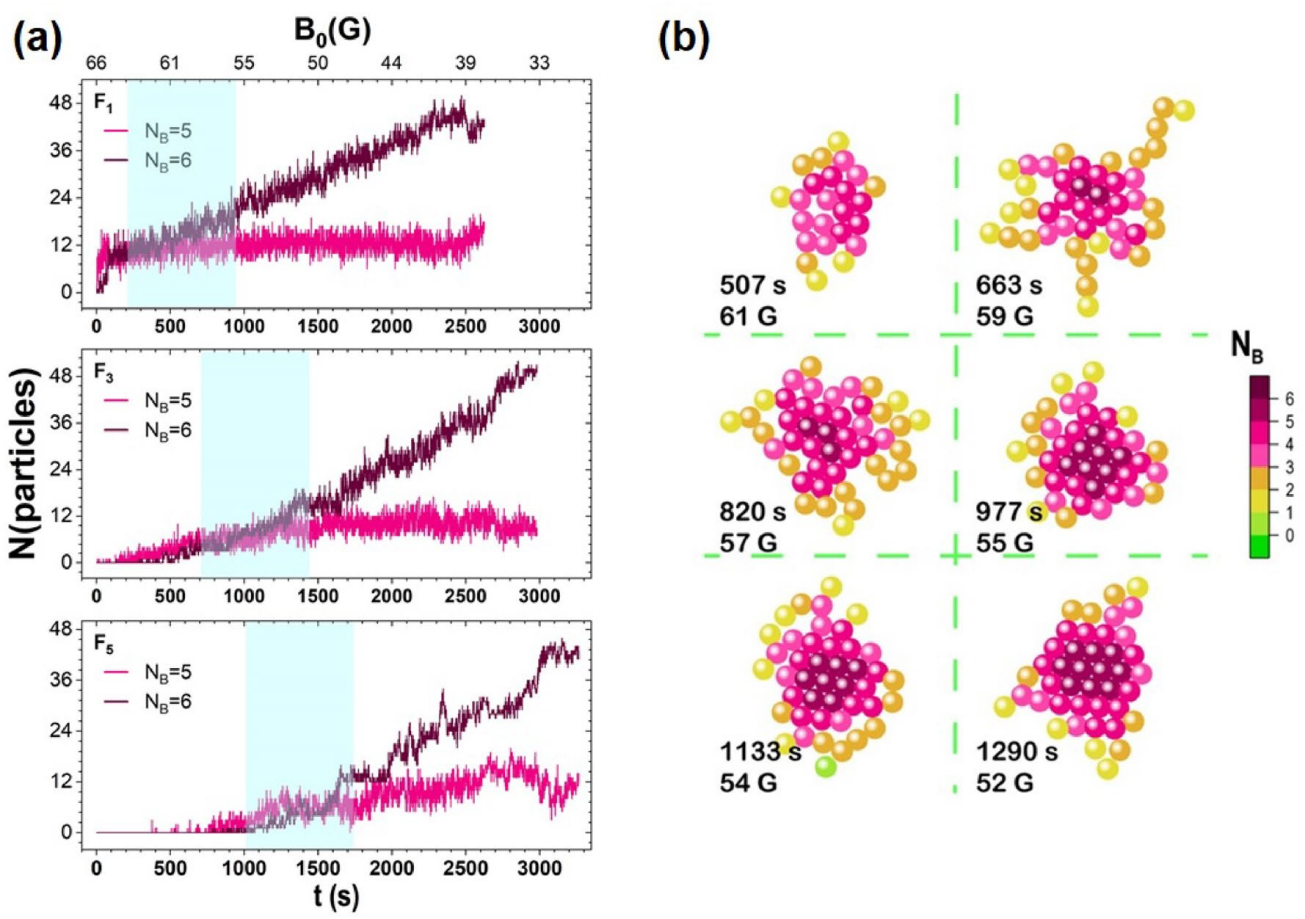
(**a**) Comparison of the populations of particles with five and six neighbours for three different cases. (**b**) A sequence of images showing the evolution from an amorphous configuration to the ordered configuration in the aggregate centre for case $F_3$.

Figure 8a shows a comparison of the populations of particles with five and six neighbours. At the beginning of the experiment, the populations of particles with five neighbours are higher than those with six neighbours; it is characteristic of an amorphous aggregate. The second step starts at a certain time as the effective temperature decreases continuously. One of the second step’s most characteristic features is the inversion of particles’ populations with five and six neighbours. This inversion is possible because the effective temperature and the kicks of the free particles drive the aggregate to acquire the compact hexagonal arrangement. See a detailed discussion about this phenomenon in Ref.^27^. In Fig. 8a, the second step is marked with a blue stripe. In Fig. 8b, it is observed in a sequence of figures showing the inversion of the population of five and six neighbours for case $F_3$ graphically. Notice that the $N_B=6$ increases monotonously while $N_B=5$ remains practically constant along the complete crystallisation process.

**Figure 9:**
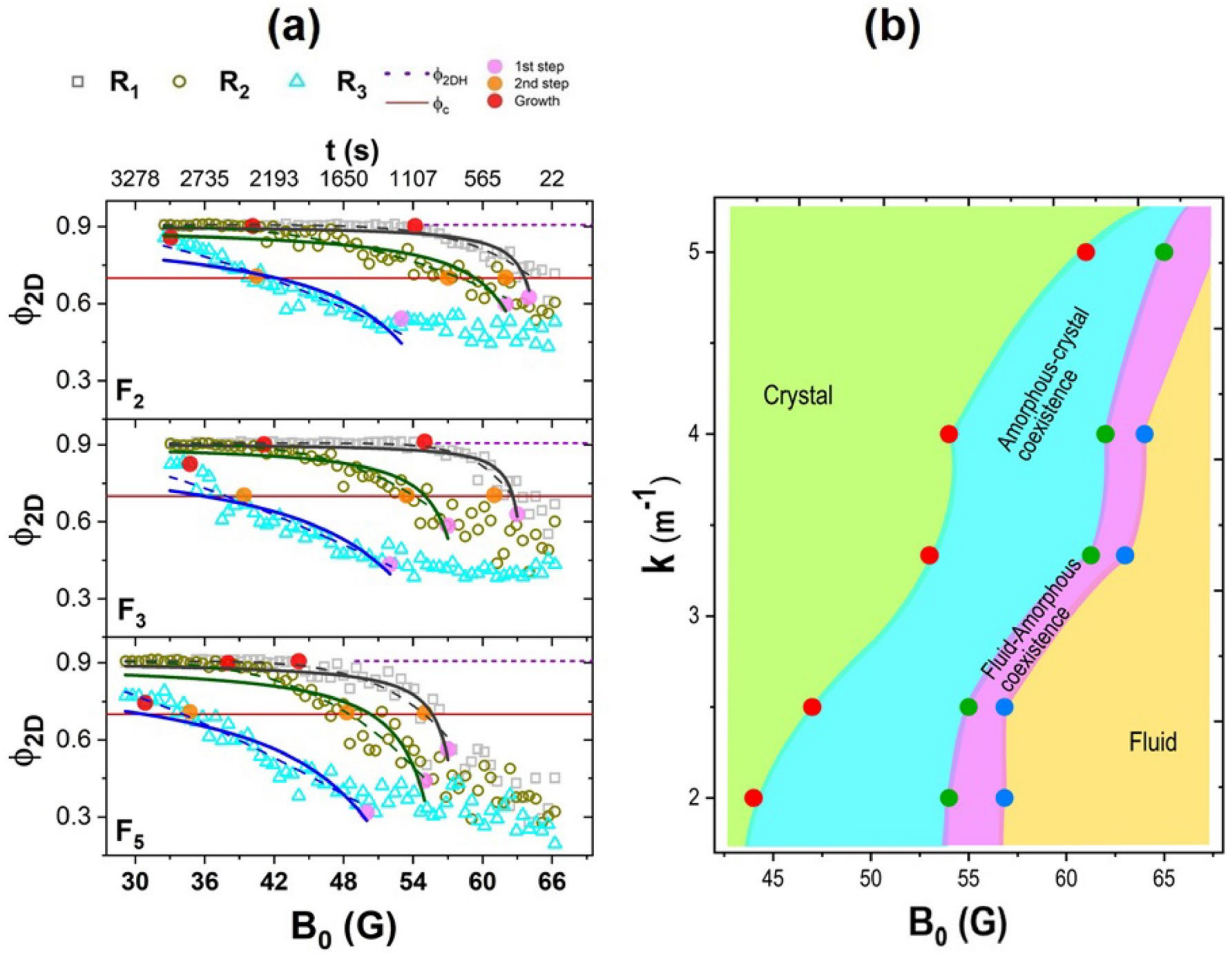
(**a**) Packing fraction, $\phi _{2D}$ as a function of the effective temperature for lenses $F_2$, $F_3$, and $F_5$ in different regions, $R_1$, $R_2$, and $R_3$. The dashed purple line corresponds to the value of the packing fraction of a perfect 2D-hexagonal close packing, $\phi _{2DH}$, and the red line corresponds to the limit value of the packing fraction of fluid hard disks, $\phi _{C}$. The pink, orange, and red circles correspond to the beginning of the first step, the second step, and the final of the second step. The black, green, and blue line correspond to the fits of the form $\phi _{2D}$=$\phi _{2DH}e^{(b/(c+B_0))}$ from the beginning of the first step (pink circle) to the final of the second step. The dashed line corresponds to a similar exponential function, $\phi _{2D}$=$\phi _{2DH}-ae^{(b/B_0)}$. (**b**) Phase diagram of curvature versus magnetic field.

For structural characterisation during the crystal growth, we use the the packing fraction, $\phi _{2D}$. Figure 9a shows the packing fraction as a function of the effective temperature for lenses $F_2$, $F_3$, and $F_5$ in three regions or areas, $R_1=4\sigma$ (grey square symbols), $R_2=7\sigma$ (green circular symbols), and $R_3=10\sigma$ (blue triangle symbols). In the $R_1$ region, the initial configuration of the particles is determined by the $\kappa$ of each lens, as it can be seen in the initial stages at high effective temperatures of Fig. 7. It is observed that the particles form a branched structure, more clearly evident as $\kappa$ decreases. In all cases, at the end of the cooling process, the particles form a compact hexagonal arrangement regardless of the lens curvature, which is evident in the behaviour of the packing fraction as a function of the effective temperature. $\phi _{2D}$ grows quickly to reach the corresponding value of a compact hexagonal arrangement, $\phi _{2DH}=0.9066$ (purple dashed line). In the intermediate region $R_2$, the packing fraction increases slower than in the central region ($R_1$) but reaches the same value, $\phi _{2DH}$. Finally, in the region $R_3$ being the outermost, the particles form a branched-type structure for a longer time; eventually, the structure started to rearrange and grow, but the value of $\phi _{2D}$ at the end of the crystallisation time is lower than that of a compact hexagonal arrangement.

The behaviour of the packing fraction also reveals that the crystallisation process occurs in two steps. Initially, the system starts in a fluid state until the formation of an aggregate starts; this is the beginning of the first step (labelled as a pink circle). This step ends when it is formed a disordered and stable aggregate. Subsequently, this aggregate rearranges and grows, and the beginning of this second step is marked with an orange circle around $\phi _{2D}=0.7$. This value is similar to the reported $\phi _{2D} \thickapprox 0.701$ for equilibrium crystallisation in a gas of hard disks^34^ (red line). The increasing of the packing fraction as the effective temperature decreases is analysed by fitting an exponential function of the type $\phi _{2D}$=$\phi _{2DH}e^{(b/(c+B_0))}$ from the beginning of the first nucleation step (pink circle) to the end of the second steps crystallisation, the fits correspond to black, green, and blue lines for the $R_1$, $R_2$, and $R_3$ region, respectively. This fit is compared with another exponential function, $\phi _{2D}$=$\phi _{2DH}-ae^{(b/B_0)}$, represented by dashed lines.

During the nucleation process, the system is affected by the curvature of the lens $\kappa$ and the magnetic field *B*. Both variables can be changed independently, at least at high temperatures. While the magnetic field behaves as the effective temperature, the lens curvature behaves as an effective pressure. Thus, $\kappa$ is the conjugate variable of the effective area occupied by the system. We have determined a phase diagram using the data of Fig. 9a and the other two experiments. Figure 9b shows the phase diagram. It can be observed a region of crystal states and a region of fluid states. Between it is observed a region of coexistence of amorphous and liquid states and another one where coexists amorphous and crystal states. For a given value of curvature, the two-step nucleation is observed while the system goes from the region of fluid to the crystal state, passing through the region of amorphous-liquid and the amorphous-crystal coexistence.

**Figure 10:**
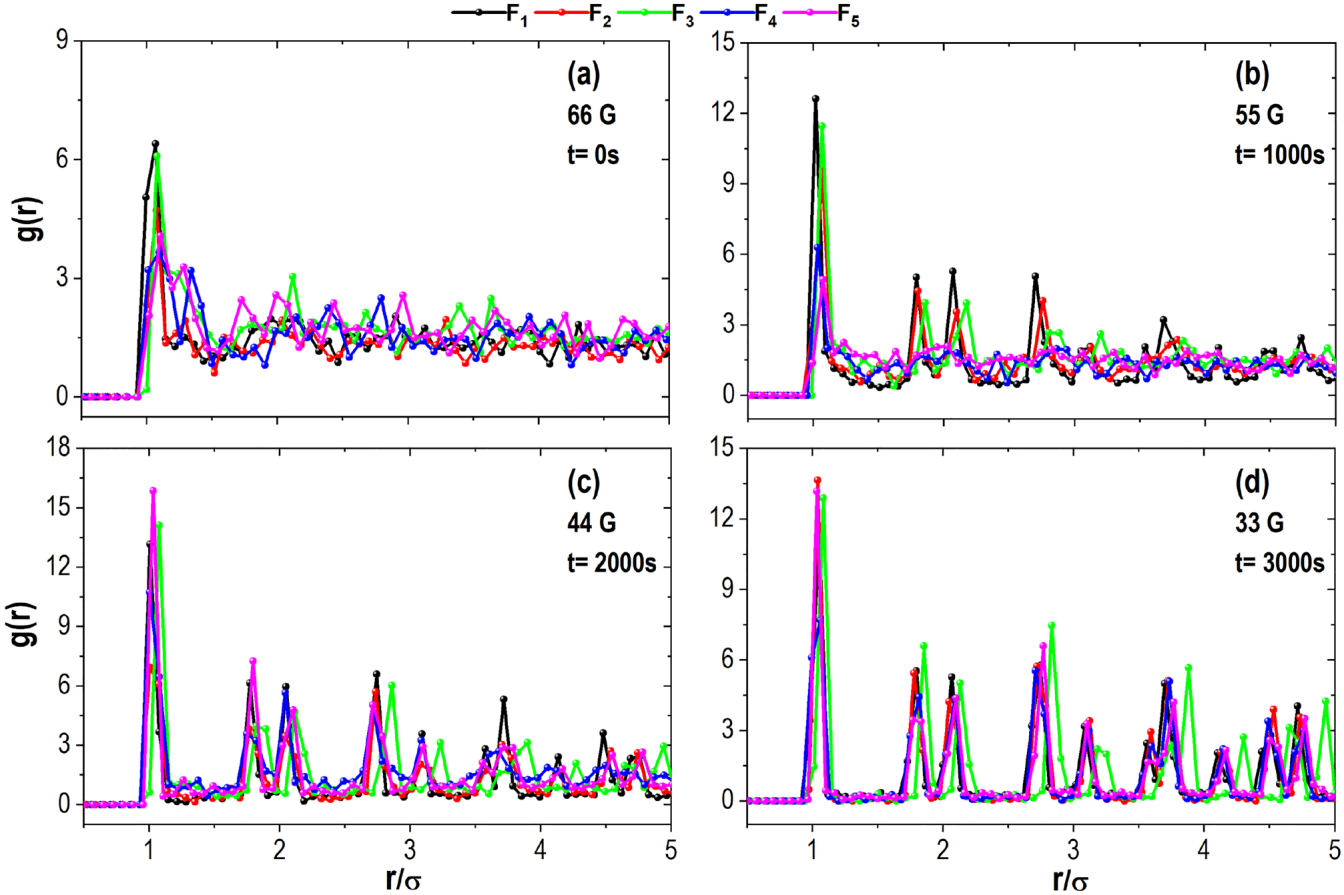
Radial distribution function, *g*(*r*) at four effective temperatures: (**a**) initial 66 G, (**b**) medium 55 G, (**c**) 44G, (**d**) and end of cooling.

Figure 10 shows the radial distribution function *g*(*r*), commonly used as a global average measure of structural order. The results are shown for four different moments during the cooling process for each lens used. Figure 10a corresponds to the beginning of the process. For lenses with higher concavity, the behaviour of the *g*(*r*) curves is similar to that of a liquid with a well-defined first peak. Figure 10b belongs to the aggregate structure after 1000 s, here the *g*(*r*) curves corresponding to the $F_1$, $F_2$, and $F_3$ lenses begin forming peaks with positions characteristic of an ordered solid state. However, for the other lenses, $F_4$ and $F_5$, the behaviour of the curves reminds that of those corresponding to a liquid. After 2000 s, the *g*(*r*) all curves have a similar shape, with more defined peaks and wells tending to zero (Fig. 10c). Although in lenses exerting lower gravitational potential, this behaviour is diminished. Figure 10d was obtained from the final structures, and it can be observed how all *g*(*r*) curves have a very similar shape. Likewise, the position of the peaks and the tendency of the wells to approach zero indicate that a compact hexagonal structure was achieved.

## Discussion and remarks

We experimentally studied crystallisation in a nonvibrating granular magnetic system settled on a concave surface in an alternating magnetic field. Under these conditions, particles behave as active particles with stochastic dynamics, where the amplitude of the magnetic field controls its effective temperature. The crystallisation was controlled by using a linear cooling profile and different parabolic potentials induced by the concavity of our different lenses. By varying the depth of the parabolic concavity, we can control the parabolic potential, which tunes the particle concentration, which controls the crystallisation rate.

It is observed that the system exhibits a two-step crystallisation process. As the concavity of the cell increases, the two-step features are more evident. The size of the stable and amorphous aggregates formed along the first step in the different lenses is more extensive when $\kappa$ is larger, and they are formed quicker as the concavity of the parabolic cell increases. Once these amorphous aggregates are formed, they evolve, grow, and reorder in the second step to form a nucleus. Crystal growth follows a similar path to nucleus formation. First, an amorphous shell is formed around the nucleus, and in the second step, it is reordering and becomes part of the crystal while the growth continues. An important remark is that the crystallisation process is truncated if the concavity depth overpasses a particular value, $\kappa =$ 6.66.

The advantage of our system is that, being a macroscopic system, we can track every single particle to determine its motion characteristics. This information lets us directly determine and calculate physical quantities to quantify and describe each process stage. Thus, this system is an excellent macroscopic model for investigating crystallisation phenomena.

Crystallisation occurs quicker the deeper the parabolic potential is. As intuitively as one expects, we have observed that aggregates are more clearly round-shaped for a larger depth of the parabolic potential. On the contrary, the structures are more branched for less parabolic potential depth, and crystallisation processes are longer. In contrast with quantities obtained as an average over the whole aggregate, such as the radial distribution function, where the local details become masked, the orientational order parameter is a suitable quantity to characterise the system’s local and global structural features. For instance, the orientational order parameter allows visualising local phases. Of course, this is particularly useful for agglomerates containing inhomogeneities formed by a few particles.

There are different possibilities to extend this study. One can combine the depth and form of the confinement potential with the cooling profile to optimise the conditions to produce homogeneous and heterogeneous crystallisation. Also, one might search for the role of the fluctuations caused by the effective temperature of the system and the kicks of the free particles on the border region of the disordered aggregate in the reordering and crystallisation growing process.

## Methods

**Figure 11:**
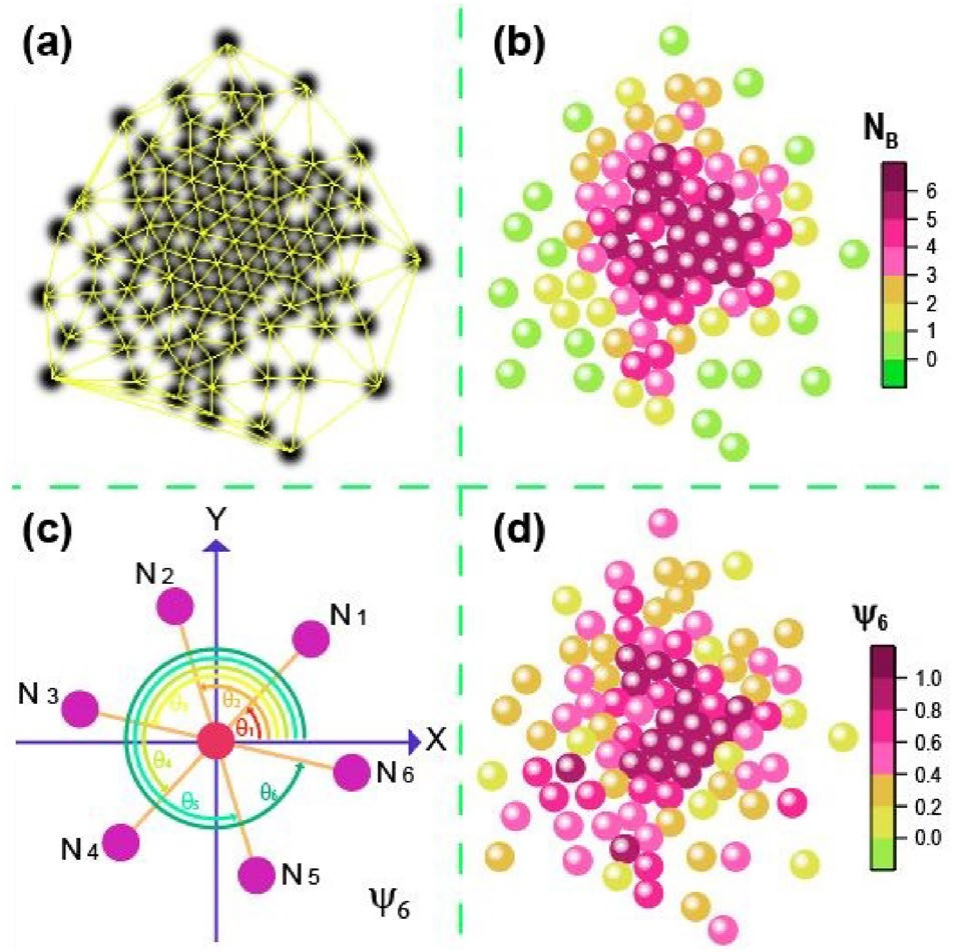
(**a**) Delaunay triangulation of the aggregate, (**b**) number of bonded neighbours, $N_B$ where the colour scale indicates the number of neighbours, (**c**) scheme of the angles that are formed by the neighbours of a particle, (**d**) the sixth orientational order parameter, $\Psi _6$.

To determine the structural characteristics of the system, we mainly use the sixth orientational order parameter $\Psi _{6}$ and the number of neighbouring particles in contact with each particle $N_{B}$. These parameters are obtained as follows. For a given particle configuration, particles are first detected and separated from each other by using ImageJ. Then, the Delaunay–Voronoi of ImageJ plugin was used to generate the Delaunay triangulation. Figure 11a shows the Delaunay triangulation for a typical configuration. The triangulation lets us determine the nearest neighbours $N_i$ of each *i* particle forming the aggregate. Using this information, we determined the number of nearest neighbours in contact with that *i* particle $N_{Bi}$; it is considered that a neighbour is in contact when it is closer than $r=1.1\sigma$ distance. Figure 11b shows the number of neighbours in contact for each particle; the colour scale indicates that the particles with a higher $N_{Bi}$ are coloured in purple, while particles with no nearest neighbours in contact are coloured in green. We could consider a scheme for each particle as shown in Fig. 11c. A central particle, coloured in red, and its nearest neighbours coloured in purple particles, each forming an angle with the x-axis. Using the information of the angles, the sixth orientational order parameter, $\Psi _{6}$ is obtained from the expression:

1 $$\begin{aligned} \Psi _{6}=\frac{1}{N_{i}}\Bigg |\sum _{j=1}^{N_{i}}\exp ({\text {i}6\theta _{ij}})\Bigg |, \end{aligned}$$

where $\theta _{ij}$ is the angle between the line formed by the reference particle *i* and neighbour *j* and the x-axis, and $N_{i}$ is the number of nearest neighbours determined based on the Delaunay triangulation^27^. Figure 11d shows the value of $\Psi _{6}$ on a colour scale, where red represents those particles with $\Psi _{6}=1$ whose neighbours form an ordered arrangement compatible with the compact hexagonal arrangement. Conversely, those particles that are not part of any structure or are completely disordered, $\Psi _{6}$, are far from the unity and are represented in green.

## Data Availability

The data-sets used and/or analysed during the current study available from the corresponding author on reasonable request.
